# Serum VEGF levels are related to the presence of pulmonary arterial hypertension in systemic sclerosis

**DOI:** 10.1186/1471-2466-9-18

**Published:** 2009-05-09

**Authors:** Andriana I Papaioannou, Epaminondas Zakynthinos, Konstantinos Kostikas, Theodoros Kiropoulos, Angela Koutsokera, Athanasios Ziogas, Athanasios Koutroumpas, Lazaros Sakkas, Konstantinos I Gourgoulianis, Zoe D Daniil

**Affiliations:** 1 Department of Respiratory Medicine University of Thessaly School of Medicine, University Hospital of Larissa, Larissa 41110, Greece; 2 Department of Intensive Care University of Thessaly School of Medicine, University Hospital of Larissa, Larissa 41110, Greece; 3 Department of Internal Medicine, University of Thessaly School of Medicine, University Hospital of Larissa, Larissa 41110, Greece

## Abstract

**Background:**

The association between systemic sclerosis and pulmonary arterial hypertension (PAH) is well recognized. Vascular endothelial growth factor (VEGF) has been reported to play an important role in pulmonary hypertension. The aim of the present study was to examine the relationship between systolic pulmonary artery pressure, clinical and functional manifestations of the disease and serum VEGF levels in systemic sclerosis.

**Methods:**

Serum VEGF levels were measured in 40 patients with systemic sclerosis and 13 control subjects. All patients underwent clinical examination, pulmonary function tests and echocardiography.

**Results:**

Serum VEGF levels were higher in systemic sclerosis patients with sPAP ≥ 35 mmHg than in those with sPAP < 35 mmHg (352 (266, 462 pg/ml)) vs (240 (201, 275 pg/ml)) (p < 0.01), while they did not differ between systemic sclerosis patients with sPAP < 35 mmHg and controls. Serum VEGF levels correlated to systolic pulmonary artery pressure, to diffusing capacity for carbon monoxide and to MRC dyspnea score. In multiple linear regression analysis, serum VEGF levels, MRC dyspnea score, and D_LCO _were independent predictors of systolic pulmonary artery pressure.

**Conclusion:**

Serum VEGF levels are increased in systemic sclerosis patients with sPAP ≥ 35 mmHg. The correlation between VEGF levels and systolic pulmonary artery pressure may suggest a possible role of VEGF in the pathogenesis of PAH in systemic sclerosis.

## Background

Systemic sclerosis (SSc) is a chronic connective tissue disease characterised by excessive collagen deposition in the skin and the internal organs and also by immunologic abnormalities and vascular injury [1]. Pulmonary complications are very common in patients with SSc [2]. The most frequent and serious pulmonary complication of SSc is pulmonary hypertension (PH) which can occur in the absence or presence of interstitial lung disease [3]. Clinically significant pulmonary arterial hypertension (PAH) affects 15–20% of scleroderma patients and results in dyspnea, impaired exercise tolerance, and a high risk of death [4,5]. The pathogenesis of SSc remains unclear and therapeutic interventions are currently limited to symptomatic relief and treatment of complications.

Vascular endothelial growth factor (VEGF) is an endothelial cell-specific mitogen and a potent angiogenic peptide which is secreted by a variety of cell types [6]. VEGF has been shown to be implicated in several physiological and pathological processes that require proliferation of endothelial cells[7]. Serum VEGF concentrations are elevated in many collagen diseases including SSc [8]. VEGF levels correlate with pulmonary function tests in several lung disorders such as asthma[9,10], chronic obstructive pulmonary disease (COPD)[10,11], and diffuse parenchymal lung diseases[10,12]. It has also been reported that VEGF plays an important role in the development of pulmonary hypertension[13,14] and is strongly expressed in the normal pulmonary circulation and within the plexiform lesions of primary pulmonary hypertension[15].

The aim of this study was to determine serum VEGF levels in patients with SSc and to evaluate whether those levels differ between patients with and without PAH not secondary to interstitial lung involvement of the disease. Furthermore we evaluated the relationship between VEGF levels and several clinical and functional parameters of the patients.

## Methods

### Patients

Forty patients with SSc (33 female, mean age 56.75 ± 12.5 years) and 13 healthy age-matched non smokers control subjects, with no history of lung disease and normal pulmonary tests, were included in the study. The diagnosis of SSc in all patients was based on the Preliminary Criteria for the Classification of Systemic Sclerosis[16]. All patients underwent high resolution computed tomography (HRCT) of the chest and pulmonary function tests (PFTs). In a separate visit all patients underwent an echocardiography by one experienced cardiologist (E.Z.).

Patients with conditions that could affect serum VEGF levels or sPAP were not included in the study. Therefore, we excluded patients with a smoking history (current smokers or ex smokers), patients with a history of coronary artery disease as well as subjects with diffuse left ventricular (LV) systolic dysfunction (Ejection Fraction <55%) or segmental abnormalities revealed by echocardiography. Moreover, we excluded patients with mitral or aortic valve disorder. Finally, we did not include patients in which a satisfactory envelope of tricuspid regurgitation (TR) could not be detected by Doppler even with the use of infusion of agitated saline.

Patients who received any kind of medication for pulmonary hypertension or had a known history of arterial hypertension receiving antihypertensive drugs, except calcium channel blockers used for Raynaud's symptoms were not included in the study. We also excluded patients with pattern consistent with interstitial lung disease (ILD) (i.e. subpleural opacities, parenchymal bands, thickened interlobular septae, an irregular pleural interface, honeycomb lung) in high resolution computed tomography, as those patients were prone to develop PH secondary to chronic respiratory disease.

Two experienced rheumatologists (L.S. and A.Z.) took a detailed clinical history and performed thorough physical examination. Disease duration was defined as duration of skin involvement. Abnormal chest sounds, finger ulcers, and total skin score were recorded for all patients. Dyspnea was evaluated with the Medical Research Council (MRC) dyspnea score[17] Blood samples were obtained from all patients and controls in order to measure serum VEGF levels. All examinations and tests in each patient were performed within a week.

The study protocol was approved by the local ethics committee and all patients gave written informed consent.

### Pulmonary Function Tests

Pulmonary function tests (PFTs) included measurement of FEV_1_, FVC, FEV_1_/FVC ratio, total lung capacity (TLC), residual volume, diffusing capacity for carbon monoxide (DL_CO_) and diffusing capacity for carbon monoxide adjusted for alveolar volume (DL_CO_/V_A_). TLC and RV were measured by the single breath helium dilution method with a commercially available system (Master Screen, Erich Jaeger GmbH, Wuerzburg, Germany). DL_CO _was assessed by means of the single breath method with the patient in the sitting position. Lung function measurements were expressed as percentages of predicted values. Tests were performed according to the American Thoracic Society guidelines[18] by the same technician in order to ensure the consistency of the results. In all patients, arterial blood samples were taken for the measurement of PaO_2 _and PaCO_2 _using a commercially available blood gas analyser (model 1630; Instrumentation Laboratories, Milan Italy)

### Dyspnea

Patients' dyspnea was assessed with the modified (5-point) Medical Research Council (MRC) dyspnea score that consists of five questions asked to patients to assess their breath discomfort according to the limitations they are experiencing secondary to shortness of breath. Scores on the modified Medical Research Council (MMRC) dyspnea scale can range from 0 to 4, with a score of 0 indicating the absence of dyspnea and 4 indicating that the patient is too breathless to leave the house or becomes breathless when dressing or undressing [17].

### High resolution computed tomography (HRCT)

The HRCT examination of the chest was performed within a 1-week interval from the pulmonary function tests and collection of blood samples. The CT scans were performed using either a Somaton HiQ or a Somaton Plus scanner (Siemens, Erlanger, Germany). Scans were performed with 1–1.5 mm section thickness and a 1–2 s scanning time during breath holding at end inspiration. Films were read blindly by a radiologist with expertise in HRCT and the presence of subpleural opacities, parenchymal bands, thickened interlobular septae, irregular pleural interface or honeycombing was recorded.

### Echocardiography

All patients underwent transthoracic echocardiogram by a single investigator (E.Z.) who was blinded to the results of the biochemical analyses and pulmonary function tests. M-mode, two-dimensional colour Doppler and Tissue Doppler imaging (TDI) were performed using standard methodology and commercially available equipment (GE Medical Systems-Vivid 3, Milwaukee, WI, USA, with a 1.5–3.6 MHz transducer)

Tricuspid regurgitant flow (TR) was identified by colour flow Doppler and the peak tricuspid regurgitant velocity was measured by continuous wave Doppler and transtricuspid gradient was assessed, based on the modified Bernoulli equation[19]. The highest velocity obtained from multiple views was used. Agitated saline was used to enhance suboptimal Doppler signals[20]. The diameter of the inferior vena cava and its respiratory variation were used to estimate right atrial pressure which was added to the transtricuspid gradient in order to estimate sPAP.

The mitral flow pattern was analysed using the pulsed-wave Doppler technique, with the sample volume located between the tips of the mitral leaflets. TDI was analysed with sample volume located at the lateral site and the inerventricular septum of the mitral annulus in the four chamber view from the apical window. All results were the average of at least three different beats.

Mild or moderate TR was found in all patients allowing a quite good Doppler envelope. Although right ventricular (RV) function was not in exclusion criteria, all patients had fairly good systolic function without RV dilation; yet, the interventricular septum performed a normal movement i.e. without bulging towards the left ventricle. All patients were in sinus rhythm; heart rate was less than 100/min during echo measurements. sPAP over 35 mmHg was used as a cut-off value for the presence of pulmonary hypertension [21,22].

### Blood samples

Blood samples were collected from all patients and control subjects during routine clinical procedures. Blood samples were centrifuged in 1500 g for 10 min at 4°C and serum was collected and frozen in -70°C until measurement. Serum VEGF levels were measured with a commercially available enzyme immunosorbent assay kit (Biosource Europe S.A.; Nivelles; Belgium) according to the protocol of the manufacturer. The lower limit of detection for VEGF was <5 pg/ml.

### Statistical analysis

Comparisons between patients and controls as well as between patients with and without PAH were performed using a Kruskal-Wallis test and are presented as the median (25^th ^to 75^th ^percentile) as variables were not normally distributed. Data were analysed using Spearman's correlation coefficient for variables that were not normally distributed and Pearson's correlation coefficient for variables which were normally distributed. In the multivariate analysis, a multiple linear regression model was created using systolic pulmonary artery pressure as the dependent variable and age, gender, disease duration, total skin score, MRC dyspnea scale and serum VEGF concentrations as independent variables. A p value of <0.05 was considered to be statistically significant. Analysis was performed using the SPSS 12 statistical package (SPSS, Chicago, IL).

## Results

**Figure 1:**
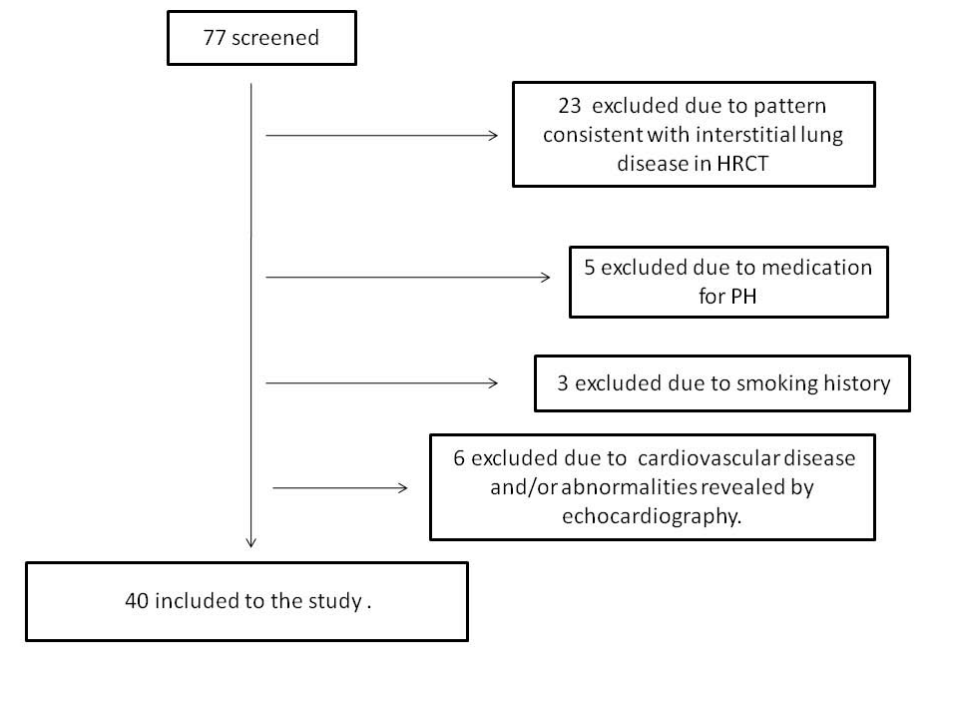
**Flow chart of the study participants**.

A flow chart of the study participants is shown in Figure 1. Demographic characteristics of the evaluated subjects are presented in Table 1. The median disease duration for the group of our patients was 6 years (range 1–21 years). Twenty four of our patients had disease duration ≤ 5 years. Skin lesions were present in all patients. However, during the time that the study was performed only nine patients had finger ulcers. The median total skin score of our study group was 17.5 (range 1–48). Systolic pressure in the pulmonary artery ≥ 35 mmHg was found in 20 patients.

**Table 1:** Demographic Characteristics and summary of pulmonary function tests of the study participants*

**Variables**	**Systemic Sclerosis**		**Controls**	**p value**
	**sPAP < 35 mmHg**	**sPAP ≥ 35 mmHg**		
Participants, No.	20	20	13	
Female/Male	16/4	17/3	10/3	
Age, ys	55.0 ± 14.0	58.4 ± 10.7	55.3 ± 13.4	0.379
FEV_1 _(%predicted)	83.9 ± 12.5	87.8 ± 18.6	99.6 ± 5.3	0.425
FVC (%predicted)	84.1 ± 14.3	86.6 ± 22.2	99.0 ± 3.2	0.570
FEV_1_/FVC	85.6 ± 9.4	85.5 ± 6.9	76.5 ± 2.9	0.882
DL_CO _(%predicted)	89.9 ± 13.4	60.0 ± 21.8	101.0 ± 4.0	<0.0001
DL_CO_/V_A _(%predicted)	88.6 ± 19.9	77.6 ± 16.3	102.3 ± 3.5	0.033
PO_2 _(mmHg)	81.8 ± 5.0	75.5 ± 12.3	94,8 ± 1.9	0.561
PCO_2 _(mmHg)	38.6 ± 3.5	37.7 ± 5.8	38.8 ± 1.2	0.942

* Values are given as the mean ± SD unless otherwise indicated. sPAP: systolic pressure in the pulmonary artery, FEV_1_: Forced Expiratory Volume in 1 sec, FVC: Forced expiratory Vital Capacity, DL_CO_: Diffusing Capacity of the lungs for Carbon Monoxide, V_A_: alveolar volume.

### Serum VEGF levels

**Figure 2:**
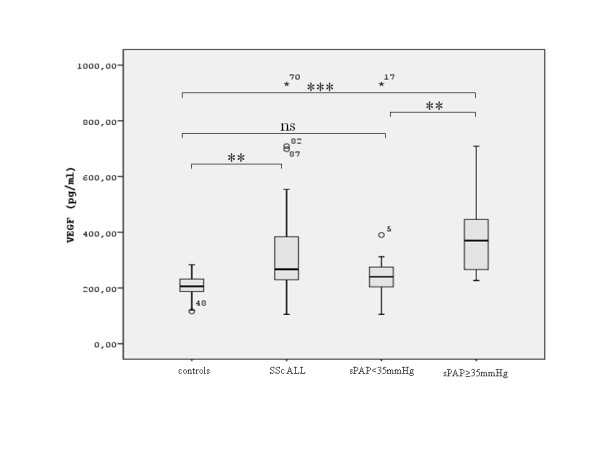
**Serum VEGF levels in patients with SSc and in healthy controls**. Data are shown as box plots, with upper and lower quartiles shaded. All: all patients with SSc, sPAP < 35 mmHg: patients with sPAP < 35 mmHg, sPAP ≥ 35 mmHg: patients with sPAP ≥ 35 mmHg. **:p < 0.01, ***:p < 0.001, ns: non significant.

Serum VEGF levels in patients with SSc were higher than in controls (267 (228, 387) pg/ml vs. 192 (169, 228) pg/ml, respectively; p < 0.01). Patients with SSc and sPAP ≥ 35 mmHg had significantly higher serum VEGF levels than those without with SPAP < 35 mmHg (352 (266–462)pg/ml vs. 240 (201–275).pg/ml respectively; p < 0.01) (Figure 2). Patients with SSc and sPAP ≥ 35 mmHg had higher serum VEGF levels than controls (352 (266–462) pg/ml vs. 192 (169, 228) pg/ml, respectively; p < 0.01) (Figure 2). No significant difference was found between patients with SSc with sPAP < 35 mmHg and controls (p > 0.05).

### Correlations of serum VEGF levels

**Figure 3:**
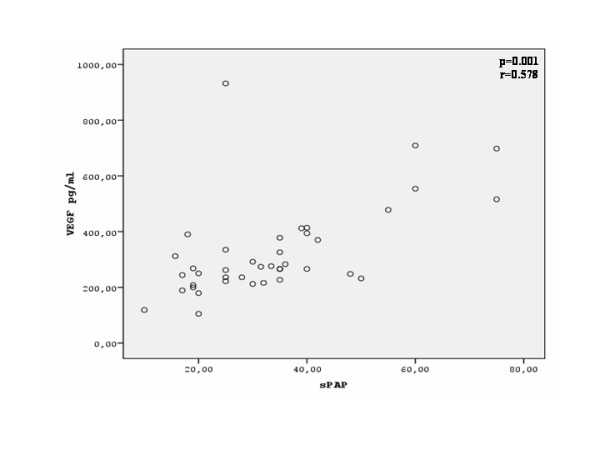
**Correlation between serum VEGF levels and systolic pulmonary artery pressure (sPAP, mmHg) in patients with SSc**.

**Figure 4:**
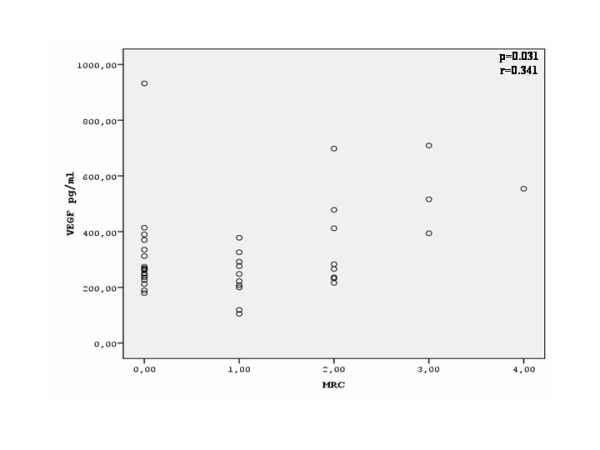
**Correlation between serum VEGF levels and MRC dyspnea score in patients with SSc**.

**Figure 5:**
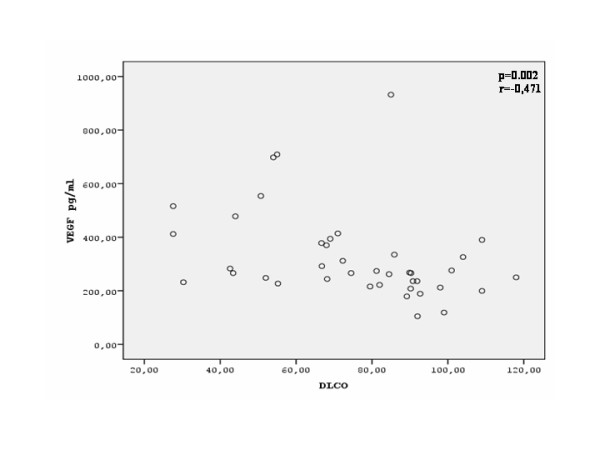
**Correlation between serum VEGF levels and D_LCO _(% predicted) in patients with SSc**.

There was a significant correlation between serum VEGF levels and sPAP (r = 0.578, p = 0.001) in patients with SSc (Figure 3). Serum VEGF levels also presented a significant correlation with the MRC dyspnea score (r = 0.341, p = 0.031) (Figure 4). Finally, serum VEGF levels presented a significant negative correlation to the diffusing capacity for carbon monoxide (D_LCO_) (r = -0.471, p = 0.002) (Figure 5) There was no statistically significant correlation between serum VEGF levels, and disease duration, blood gases, FEV_1_, FVC, FEV_1_/FVC ratio or total skin score in the group of our patients.

### Correlations of sPAP with clinical and functional parameters

sPAP significant correlated to the patients dyspnea (measured by the MRC dyspnea scale) and to the diffusing capacity for carbon monoxide (r = 0.662, p < 0.001 and r = -0.722, p < 0.001 respectively). There was no statistically significant correlation between serum sPAP levels, and arterial blood gases, FEV_1_, FVC, or FEV_1_/FVC ratio in the group of our patients.

### Multivariate analysis for determinants of sPAP

In the multiple linear regression analysis, sPAP was used as dependent variable whereas age, gender, disease duration, total skin score, MRC dyspnea score, D_LCO _and serum VEGF concentrations were used as independent variables. The only independent determinants of sPAP (R^2 ^= 0.724) were serum VEGF levels (p = 0.020, β = 0.295), D_LCO _(p = 0.003, β = -0.454) and MRC dyspnea score (p = 0.018, β = 0.344). (Table 2)

**Table 2:** Multivariate analysis for determinants of sPAP

**Variable**	B*	**SE**	**β^†^**	**(95% CI for B)**	**p**
Age	0.015	0.137	0.011	(-0.267, -0.297)	0.915
Gender	-4.258	4.343	-0.107	(-13.184, 4.669)	0.336
Disease duration	-0.282	0.358	-0.093	(-1.019, 0.454)	0.438
MRC dyspnea score	4.996	1.987	0.344	(0.913, 9.080)	0.018
Total skin score	-0.083	0.177	-0.053	(-0.446, 0.280)	0.643
D_LCO_	-0.316	0.095	-0.454	(-0.511, -0.122)	0.003
Serum VEGF	0.027	0.011	0.295	(0.005, 0.050)	0.020

*Unstandardised coefficient; ^†^standardised coefficient; SE: standard error, Adjusted R^2 ^= 0.724

## Discussion

In the present study we have shown that serum VEGF levels were elevated in patients with SSc and sPAP ≥ 35 mmHg compared to patients with SSc and sPAP < 35 mmHg and healthy controls. In addition, serum VEGF levels were positively correlated to the levels of sPAP and the degree of dyspnea as expressed by the modified MRC scale and were negatively correlated to the diffusing capacity for carbon monoxide (D_LCO_). Finally, in the multivariate analysis, we have shown that serum VEGF levels were a determinant of sPAP, suggesting a possible role for VEGF in the pathogenesis of PAH in patients with SSc.

The pathogenesis of SSc involves interplay between obliterative vasculopathy in multiple vascular beds, inflammation, autoimmunity and progressive fibrosis [1,2,23]. Vascular injury and activation are the earliest and possibly primary events in the pathogenesis of SSc [2]. Among the manifestations of SSc-associated vasculopathy, PH is the most severe and life-threatening one. Angiogenic factors are likely to be involved in the initiation of endothelial cell dysfunction and the development of pulmonary hypertension [24] VEGF and its receptors flt-1 and flk-1 are expressed in the plexiform lesions in the lungs of patients with severe PH. In addition, overexpression of VEGF produces structures that resemble plexiform lesions [25]. It is well known that chronic hypoxia increases lung tissue VEGF expression and that VEGF is likely a modulator of chronic hypoxia-induced pulmonary vascular remodelling [26]. It has also been reported that VEGF is increased in rats with hypoxia and monocrotaline-induced PH and vascular remodelling [26,27]. These findings have led to the hypothesis that VEGF may contribute to the pathogenesis of PH by stimulating dysregulated angiogenesis [25,28].

Our data are in agreement to previous studies which have reported that serum VEGF levels are higher in patients with SSc compared to controls [29]. It is known that VEGF levels are increased under hypoxic conditions such as reduced blood flow and reduced partial oxygen pressure levels [6,30]. It is also known that systemic sclerosis is associated with reduced capillary density which leads to a reduced blood flow and thus to tissue ischemia and to clinical manifestations such as fingertip ulcers [29]. However, in our group of patients, no significant correlation was found between serum VEGF levels and either the severity of skin lesions, expressed as the total skin score, or the presence of finger ulcers. A plausible explanation for this finding could be the fact that tissue hypoxia described in SSc is not limited to the skin but also involves several internal organs [2,23], which may contribute to the overall elevation of VEGF levels in the serum of these patients. On the other hand, our data suggest that serum VEGF levels are higher in patients with SSc and sPAP ≥ 35 mmHg and do not differ between patients with SSc and sPAP < 35 mmHg and controls. This fact leads to the hypothesis that increased VEGF levels might be related to the presence of elevated pressure in the pulmonary artery rather than to the disease on its own. Our findings may support the hypothesis that elevated serum VEGF levels might reflect an increase in VEGF production at sites of vascular injury due to tissue hypoxia[25,28] which enhances dysregulated angiogenesis in the pulmonary vasculature leading to the development of PAH. However, as it can be observed, there is an overlap in the sVEGF levels between patients with sPAP ≥ 35 mmHg and patients with sPAP < 35 mmHg. In this study we excluded patients with conditions that could affect sVEGF levels and for that reason the causes for this overlap are difficult to be determined. Further studies are needed to determine whether patients with low sPAP and high sVEGF will develop PAH in the future.

In contrast to the suggestion that VEGF plays a significant role in the pathogenesis of PH, a recent report indicated that VEGF likely plays an important role in the maintenance of normal pulmonary vascular structure [31]. This report suggested that VEGF is important in attenuating the development of PH possibly by protecting endothelial cells from injury and apoptosis [31]. It has been proposed that VEGF is a potential mediator of endothelial cell proliferation and thus it may act as a protective mechanism against pulmonary vascular injury and remodelling [13]. It has also been reported that inhibition of VEGF receptor flk-1 in animal models caused pulmonary hypertension characterized by thickening of the medial layer of pulmonary arteries in normoxic conditions [32]. Additionally, severely hypoxic rats which were treated with an flk-1 inhibitor, developed PH which was accompanied by a marked increase in endothelial cell proliferation in the pulmonary artery [32]. This probable protective role of VEGF during the development of PH can also be supported by the fact that VEGF increases local eNOS expression and stimulates NO release from vascular endothelium [33]. Therefore, an alternative interpretation of the increased serum VEGF levels in patients with SSc and sPAP ≥ 35 mmHg, could be that the elevation of sPAP might by itself be the cause of the induction of VEGF synthesis and release from the pulmonary vasculature, in an attempt to blunt the development of vascular remodelling in the pulmonary vessels[33].

Our data have also shown a significant correlation between serum VEGF levels and dyspnea as expressed by the MRC dyspnea score. In patients with SSc, dyspnea occurs either due to the involvement of the lung parenchyma or due to the development of PH, both potentially resulting in oxygen desaturation especially during exercise [2]. As previously mentioned, under hypoxic conditions VEGF expression normally increases. However, no correlation was found between VEGF levels and the values of the arterial blood gases in our patients. Furthermore, arterial PO_2 _did not differ significantly between the two groups of our patients. A plausible explanation for the absence of a significant correlation could be the fact that blood gases were collected and measured at rest, whereas the greater degree of hypoxia occurs during exercise and MRC dyspnea score expresses dyspnea in activity.

Finally we have found a significant correlation between serum VEGF levels and D_LCO_. In patients with SSc an isolated reduction of D_LCO_with normal lung volumes is indicative of PAH [34]. According to the fact that reduced D_LCO _in PAH occurs due to vascular damage, the correlation between serum VEGF levels and D_LCO _provides further implications that VEGF might play a role in the pathogenesis of PAH in SSc.

There are certain limitations in the present study. First, we did not perform right heart catheterization to confirm sPAP measurement assessed by echocardiography. However, Doppler echocardiography is a reliable and reproducible means of non-invasive assessment of sPAP [19,35] that has been employed to detect PH in patients with connective tissue diseases [36]. In our study we excluded patients with left ventricular (LV) systolic dysfunction or known cardiac disease which could increase sPAP due to increased left ventricular filling pressures. Although LV diastolic dysfunction could contribute to the increase of sPAP due to postcapillary hypertension this does not seem possible as diastolic function assessed from transmitral Doppler flow and TDI did not differ between SSc patients with sPAP ≥ 35 mmHg and those with sPAP < 35 mmHg. This is in keeping with a large study by Aguglia et al. who did not consider the existence of SSc by itself, as an independent factor affecting LV diastolic function in SSc patients [37]. Furthermore, it is a fact that a great number of our patients had elevated estimated levels of sPAP in echocardiography. A possible explanation for that might be that the study participants were recruited from a scleroderma clinic in a tertiary hospital suggesting that they were patients who had more severe disease. Another limitation is that our analyses are based in single measurements of sPAP and serum VEGF levels which may not reflect this relationship in the course of the disease. Therefore, further prospective studies are needed to investigate whether the worsening in sPAP is followed by increases in serum VEGF levels.

## Conclusion

In conclusion, in our study we have shown that serum VEGF levels are elevated in patients with systemic sclerosis and sPAP ≥ 35 mmHg compared to SSc patients and sPAP < 35 mmHg and to healthy controls. Although the exact role of VEGF in the pathogenesis of PH and vascular remodelling remains controversial, our findings suggest that serum VEGF levels may be used as a predictor of sPAP in patients with SSc. Further studies are promptly needed in order to evaluate the importance of determining serum VEGF levels for the early detection, monitoring and prognosis of PAH in patients with SSc.

## Abbreviations

COPD: Chronic obstructive pulmonary disease; DL_CO_: Diffusing capacity for carbon monoxide; DL_CO_/V_A_: diffusing capacity for carbon monoxide adjusted for alveolar volume; FEV_1_: forced expiratory volume in one second; FVC: forced vital capacity; HRCT: High resolution computed tomography; ILD: Interstitial lung disease; LV: Left ventricular; MRC: Medical Research Council; PAH: Pulmonary arterial hypertension; PFTs: Pulmonary function tests; PH: Pulmonary hypertension; RV: Right ventricular; SSc: Systemic sclerosis; sPAP: Systolic pulmonary artery pressure; TDI: Tissue Doppler imaging; TLC: Total lung capacity; TR: tricuspid regurgitation; VEGF: Vascular endothelial growth factor.

## Competing interests

The authors declare that they have no competing interests.

## Authors' contributions

AP, KIG and ZD were involved in the study conception. EZ performed the echocardiography and the measurements of sPAP. TK performed serum VEGF measurements. LS and AK were involved in patients' examination and selection. AP, AK and KK performed the data acquisition and interpretation. AP and KK performed the statistical analysis. AP, ZD and KK prepared the manuscript. ZD and KIG were involved in revising the manuscript for important intellectual content. All authors read and approved the final manuscript.

## Pre-publication history

The pre-publication history for this paper can be accessed here:


